# Composite Ensemble Learning Framework for Passive Drone Radio Frequency Fingerprinting in Sixth-Generation Networks

**DOI:** 10.3390/s24175618

**Published:** 2024-08-29

**Authors:** Muhammad Usama Zahid, Muhammad Danish Nisar, Adnan Fazil, Jihyoung Ryu, Maqsood Hussain Shah

**Affiliations:** 1 Electrical and Computer Engineering Department, Sir Syed CASE Institute of Technology, Islamabad 04524, Pakistan; usamazahid1@ymail.com (M.U.Z.);; 2 Department of Avionics Engineering, Air University, E-9, Islamabad 44230, Pakistan; 3 Electronics and Telecommunications Research Institute (ETRI), Gwangju 61012, Republic of Korea; 4 SFI Insight Centre for Data Analytics and the School of Electronic Engineering, Dublin City University, D09 V209 Dublin, Ireland

**Keywords:** deep ensemble learning, deep learning, drone fingerprint, ensemble learning, RF fingerprinting, specific emitter identification

## Abstract

The rapid evolution of drone technology has introduced unprecedented challenges in security, particularly concerning the threat of unconventional drone and swarm attacks. In order to deal with threats, drones need to be classified by intercepting their Radio Frequency (RF) signals. With the arrival of Sixth Generation (6G) networks, it is required to develop sophisticated methods to properly categorize drone signals in order to achieve optimal resource sharing, high-security levels, and mobility management. However, deep ensemble learning has not been investigated properly in the case of 6G. It is anticipated that it will incorporate drone-based BTS and cellular networks that, in one way or another, may be subjected to jamming, intentional interferences, or other dangers from unauthorized UAVs. Thus, this study is conducted based on Radio Frequency Fingerprinting (RFF) of drones identified to detect unauthorized ones so that proper actions can be taken to protect the network’s security and integrity. This paper proposes a novel method—a Composite Ensemble Learning (CEL)-based neural network—for drone signal classification. The proposed method integrates wavelet-based denoising and combines automatic and manual feature extraction techniques to foster feature diversity, robustness, and performance enhancement. Through extensive experiments conducted on open-source benchmark datasets of drones, our approach demonstrates superior classification accuracies compared to recent benchmark deep learning techniques across various Signal-to-Noise Ratios (SNRs). This novel approach holds promise for enhancing communication efficiency, security, and safety in 6G networks amidst the proliferation of drone-based applications.

## 1. Introduction

Unmanned Aerial Vehicles (UAVs) are commonly referred to as drones, and have a wide variety of both military and civilian applications [1]. They are used in advertising, transportation, firefighting, search and rescue operations, traffic monitoring, and atmospheric studies in the civilian sector; however, they are mostly used for reconnaissance in the military. Applications and usage of drones are continuously expanding, and this poses security risks. Therefore, it is critical to determine the existence and specific role and application of each drone. Although they are not sufficiently dependable, conventional techniques, including the use of sensors, acoustic signals, and radars, are used for this purpose [2]. This study reviews the use of radar, electro-optical sensors, thermal sensors, and acoustic sensors for radio frequency (RF)-based drone detection and classification. This section looks at traditional drone detection research first, followed by RF-based studies. One typical instrument for spotting flying vehicles is a radar sensor. It is not impacted by bad weather and perceives long-range operation compared to other sensors [3]. Nevertheless, it falls short when it comes to identifying small, slowly moving objects like drones [4]. To classify surveillance radar data as birds and UAVs, a probabilistic motion model has been developed [5]. Additionally, the technique in [6] applies UAV classification utilizing Linear Frequency Modulated Continuous Wave (LFMCW) 2D surveillance radar data. According to a different study, radar sensors are generally dependable for detecting drones, notwithstanding their inefficiency in categorization [7].

On the other hand, optical sensors are an all-purpose tool for image processing. Images were first taken with optical cameras for the purpose of classifying UAVs. These images are then classified using Deep Neural Network (DNN)-based image processing techniques like Faster-RCNN [8], VGG-16 [9], and ZF-net [10], or classical methods like Histogram of Gradients (HOG) using optical cameras [11]. High-resolution cameras are necessary for all of these techniques to effectively distinguish drones from background photos. Furthermore, it could be challenging to tell UAVs apart from tiny things like birds [7]. As an alternate strategy to address this issue, the use of thermal sensors is recommended. The heat that is released by things that optical sensors are unable to see can be captured and detected by them. Panoramic thermal cameras are used in [12] to detect drones because they work well for nighttime surveillance, and can take pictures in inclement weather, such as rain, snow, fog, etc. Nevertheless, a significant drawback is their susceptibility to moisture [7].

Acoustic sensors are another kind of sensor utilized for drone detection, similar to radar and imaging sensors. These low-cost sensors have the potential to differentiate UAV noises from other noise sources. Nevertheless, noise has a significant impact on these sensors [7]. For instance, radar and acoustic sensors are combined in [13] to identify UAV rotor types. Similarly, the authors of [14] propose a Green and Low-Resource Specific Emitter Identification method (GLR-SEI) using complex networks and Fisher pruning. Evaluated with real-world ADS-B data, their method showed promising results. In [15], the authors introduced a novel Long-Tailed Specific Emitter Identification (LT-SEI) method using Decoupled Representation (DR) learning. Evaluated with real-world ADS-B data, their method demonstrated superior long-tail recognition performance. Furthermore, UAVs are detected using high-resolution cameras [16]. Using acoustic sensors in the K-Nearest Neighborhood (KNN) approach, real-time drone identification and tracking are investigated [17]. However, according to reports, UAV identification becomes challenging if the acoustic sensor is placed more than 150 m away from the UAV [18]. Drone detection has lately made use of drone-emitted radio frequency signals as an alternate method [19]. For that reason, a sizable data set called DroneRF was assembled from various drones [19]. These data set is used to detect and classify drones in the surrounding area, by different techniques.

UAV detection and classification were performed using the DNN method in recent research, keeping in view the limitations of conventional methods [20,21]. Drone signal detection and classification were studied using the DroneRF data set and the 1-DCNN method [22,23], along with 10-fold cross-validation. The work in [24] exclusively uses low-band signals, even though these studies use both low-band and high-band RF signals. For that, they also employed the XGBoost algorithm. To distinguish between various drones, the authors of [25] suggested a Deep Complex valued Convolutional Neural Network (DC–CNN)-based RF fingerprinting. They used nine alternative algorithm models and two distinct RF drone signal datasets to perform drone detection and classification. In [26], drone detection performance is examined in terms of RF signal source distance through the development of a drone detection mechanism. Five distinct drones are used in [27] to estimate the loads carried by drones up to a 200-m distance.

Drone classification is not just restricted to security and defense, as mentioned in previous paragraphs. There are plenty of applications that have emerged with the advancements in future 6G technology [28]. Drone signal classification in 6G networks offers multifaceted benefits. By accurately discerning between different drone signals, 6G networks can optimize the allocation of resources like bandwidth and power, ensuring efficient communication for drones [29]. This classification capability also bolsters security measures by distinguishing between legitimate drone activities and potential threats, enhancing airspace safety [30]. Moreover, it enables dynamic spectrum sharing, optimizing spectrum utilization, and minimizing interference, thus boosting overall network capacity and performance. Additionally, with the ability to adapt mobility management strategies based on signal classification, 6G networks ensure seamless connectivity and efficient handover between ground-based and aerial networks [31]. Furthermore, tailored Quality of Service (QoS) guarantees and optimizations for diverse drone applications can be provided, fostering the widespread adoption of drone-based services across various sectors in 6G networks [32].

Recently, deep ensemble learning-based approaches have also gained significant attention from the research community. The technique has been widely applied, and demonstrated promising results. These types of techniques not only demonstrated their potential, but also their strength in handling and finding complex patterns in the data. In [33], the authors introduced a novel framework utilizing neural network-based concepts and reduced feature vectors, combined with multiple machine learning techniques, to accurately classify mitotic and non-mitotic cells in breast cancer histology images, outperforming existing methods in accuracy and efficiency. Similarly, in [34], the authors propose DCNN-4mC, a neural network-based tool, for accurate identification of DNA N4-methylcytosine (4mC) sites, achieving superior performance across multiple species datasets compared to existing computational tools. In [35], the authors introduce m6A-NeuralTool, a computational model for efficient identification of N6-methyladenosine (m6A) sites, achieving superior accuracy compared to existing models across multiple species datasets, facilitating rapid and accurate identification of m6A modifications for biomedical research and product development. In [36], the authors present DL-m6A, a novel deep learning-based tool for identifying N6-methyladenosine (m6A) sites in mammals, demonstrating superior performance compared to existing methods across tissue-specific and full transcript datasets. This tool offers enhanced accuracy and contextual feature representation, providing valuable insights for biology experts, and is accessible via a freely available web server. In [37], the authors introduce MCSE-enhancer, a multi-classifier stacked ensemble model, which effectively identifies enhancers by integrating experimental and computational approaches. By leveraging physiochemical properties as feature descriptors and employing a stacked classifier, MCSE-enhancer outperforms previous techniques, achieving an accuracy of 81.5%, marking a notable improvement over existing models. Despite showing its strengths in machine vision, image processing, and microbiology, there is very limited research in the field of RF signal classification, especially for drone classification. This study uses the drones benchmark dataset [38] to improve the classification resilience and accuracy. More precisely, the major contributions of this manuscript are:

- Proposing a novel feature extraction technique (manual signal processing-based) to complement automatic feature extraction by deep network layers (convolution and Long Short-Term Memory [LSTM] layers).
- Implementing both automatic and manual feature fusion within the Composite Ensemble Learning (CEL) framework for enhanced pattern extraction.
- Comparing the proposed method with the state-of-the-art deep learning techniques to demonstrate its efficacy.

The rest of the paper is structured as follows. The methodology is presented in Section 2 of this article. The used data set is explained in Section 3. The experiments, results, and analysis of the study are presented in Section 4. The discussion is in Section 5, and finally, the conclusion of the paper is presented in Section 6.

## 2. Proposed Methodology

**Figure 1:**
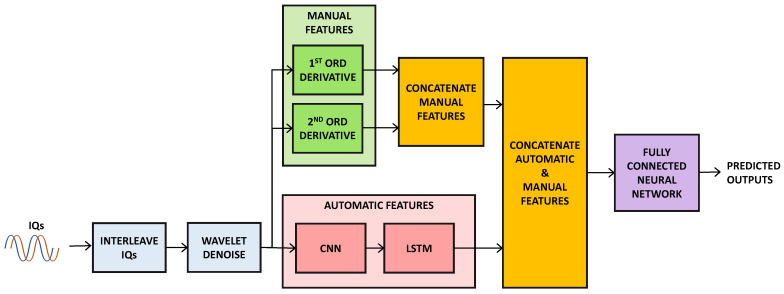
Block diagram of the proposed drone classification method.

Figure 1 is the main block diagram that demonstrates the proposed methodology. The process starts with multiple IQ signals that are multiplexed into an ordered series of real numbers, thereby making them analytically usable. This interleaving step becomes important for translating the intricate IQ data set into a structure more compatible with the deep learning-related processes.

The interleaved IQ signals are then passed through the denoise block, which executes signal filtering to enhance the signal quality and reduce variance due to the presence of background noise. This is very crucial, as it helps in increasing the efficiency of the subsequent feature extraction. For signal denoising, discrete wavelets are employed, which are very useful in denoising through multiresolution analysis, since they analyze the noise and the signal attributes at various levels of resolution. They are used to perform localized filtering, both in the time and frequency domains, which could be of immense help in correctly filtering out noises that are usually embedded, while at the same time preserving the important aspects of the signal. Also worth elaborating is the fact that wavelet-based thresholding techniques are adjusted to particular features of noise, which contributes to the improvement of the signal quality. After the denoising, the signal flows into three parallel streams for feature extraction. The first derivative subdivision determines the first derivative of the denoised signals that contain rate change data indicating large fluctuations. The second derivative branch performs the second derivative to find accelerations and other fine details of the signal in addition to the slope, while CNN and LSTM Branch use feature extraction based on CNNs and LSTMs, CNNs are designed to extract spatial features and LSTMs are used to address temporal information allowing the model to harness the information in sequential data. Features from the three branches are combined into a composite feature vector which consists of both manually engineered and automatically generated features. It is then passed through the dense layers and the final sigmoid layer to obtain the resultant composite vector. This is followed by the dense layers that add more depth to the features, and the sigmoid layer that outputs a probability for each drone class, resulting in the classification decision.

### 2.1. Signal Model and Denoising

**Figure 2:**
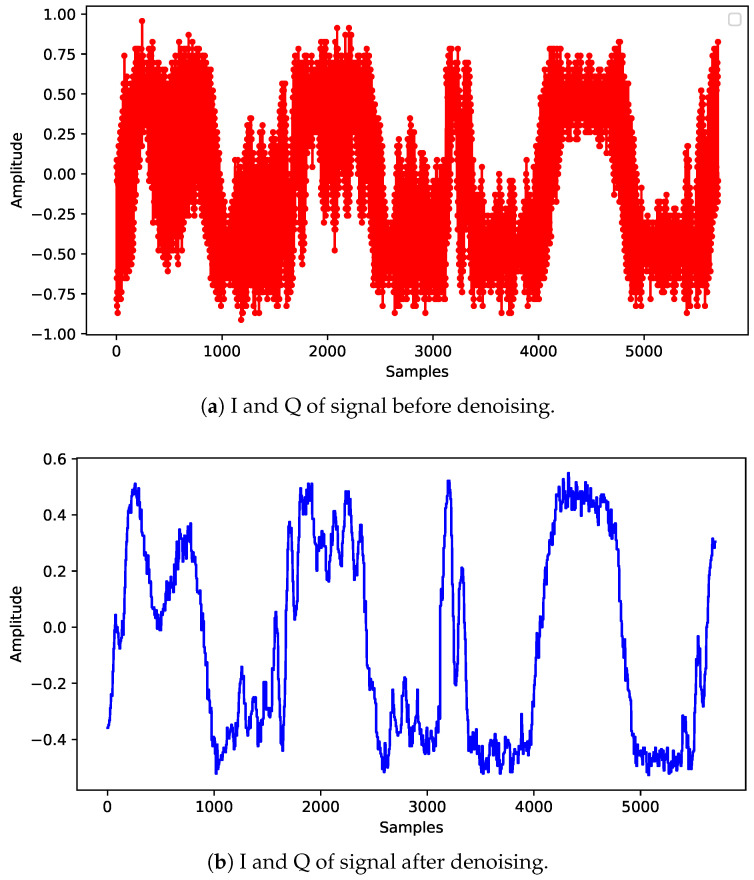
Signal samples before and after denoising.

The first step is reading time domain signals from drones using a benchmark dataset [38]. This is illustrated in Figure 2a. Signals acquired with complex IQs cannot be processed directly in the network because only real values are received at the input layer. First, the complex value is decomposed into real and imaginary values, and then the samples are interleaved to form an array of real values. This is represented by (1)

(1) $$\vec{x}\left(n\right)=\vec{s}\left(n\right)+\vec{v}\left(n\right),\;\mathrm{where}\;\vec{v}\left(n\right)∼N\left(0,1\right),$$

where $\vec{x}\left(n\right)$ is the measured noisy signal, $\vec{s}\left(n\right)$ is the original signal, and $\vec{v}\left(n\right)$ is the Gaussian noise with zero mean. The equivalent representation of (1) is given by (2)

(2) $$\vec{x}\left(n\right)=\left[x\left(1\right),x\left(2\right),\ldots ,x\left(N\right)\right].$$

The denoising of the signal is done by calculating wavelet coefficients, represented by (3)

(3) $$c\left(n\right)=\sum_{n\in Z}x\left(n\right)g_{j,k}\left(n\right),\;\mathrm{where}\;j\in N,\;k\in Z.$$

The threshold values are determined after obtaining the wavelet coefficients. We apply hard thresholding due to its simplicity, where the absolute values of all wavelet coefficients below the threshold are set to zero. The hard thresholding function is defined as (4)

(4) $$c^{\prime }\left(n\right)=\left\{\begin{array}{cc} c(n) & \mathrm{if}\;|c(n)|>T\\ 0 & \mathrm{otherwise}, \end{array}\right.$$

where

(5) $$T=\sigma \sqrt{2\log_{10}\left(N\right)},$$

and

(6) $$\sigma =\mathrm{median}\left(\frac{\left|c(n)\right|}{0.6745}\right).$$

The coefficients in the frequency domain are represented by (4). To convert back into the time domain, the inverse discrete wavelet transform is computed, obtaining (7), as illustrated in Figure 2b:

(7) $$\vec{x^{\prime }}\left(n\right)=\left[x^{\prime }\left(1\right),x^{\prime }\left(2\right),\ldots ,x^{\prime }\left(N\right)\right].$$

### 2.2. Feature Extraction

The processed signal in (7) is then split into two branches. One branch is for manual feature extraction, and the other stacked branch is in parallel for automatic feature extraction. The first and second derivatives of a time-domain signal play crucial roles in pattern extraction and analysis across diverse fields. In signal processing, the first derivative offers insights into the rate of change of the signal at each point, effectively capturing the slope or gradient of the waveform. The first derivative aids in edge detection, facilitating the delineation of boundaries between regions of interest.

(8) $${\vec{x}}^{\prime \prime }\left(n\right)=\frac{d}{dn}\vec{x^{\prime }}\left(n\right)=\frac{d}{dn}\left[x^{\prime }\left(1\right),x^{\prime }\left(2\right),\ldots ,x^{\prime }\left(N\right)\right].$$

The second derivative provides additional depth to pattern analysis by revealing curvature-related attributes of the time-domain signal. It denotes the rate of change of the slope and effectively highlights concave and convex regions within the waveform. This information aids in detecting inflection points, where the curvature changes direction, signifying potential shifts or transitions in the underlying pattern. Moreover, the second derivative enhances the robustness of pattern extraction algorithms by facilitating noise reduction. Focusing on the curvature of the signal helps filter out high-frequency noise, thereby improving the SNR and enhancing the accuracy of pattern recognition of drone signals.

(9) $${\vec{x}}^{\prime \prime \prime }\left(n\right)=\frac{d^{2}}{dn^{2}}\vec{x^{\prime }}\left(n\right)=\frac{d^{2}}{dn^{2}}\left[x^{\prime }\left(1\right),x^{\prime }\left(2\right),\ldots ,x^{\prime }\left(N\right)\right]$$

Then, we fuse both feature vectors to form our final manually crafted feature vector, represented as (10).

(10) $${\vec{x}}_{\mathrm{mf}}\left(n\right)=\left[{\vec{x}}^{\prime \prime }\left(n\right),{\vec{x}}^{\prime \prime \prime }\left(n\right)\right]$$

In the domain of pattern recognition and automatic feature extraction, convolutional layers are instrumental due to their proficiency in discerning spatial patterns within the input data. Through convolution operations employing trainable filters, CNNs adeptly detect intricate features like edges, textures, and shapes in images or sequences. By stacking multiple convolutional layers, CNNs progressively extract hierarchical representations, with each layer capturing increasingly abstract and complex patterns. This hierarchical approach facilitates the identification of high-level features by building upon lower-level representations, thereby enabling CNNs to recognize diverse and nuanced patterns present in the data. Additionally, CNNs have a feature called translation invariance, which enables them to recognize patterns regardless of where they are located in the input. This is an important feature for tasks like object detection and image localization.

Long Short-Term Memory (LSTM) layers, on the other hand, are extremely important for modeling sequential data and deriving temporal dependencies. LSTM networks are superior to typical Recurrent Neural Networks (RNNs) at capturing long-range dependencies and maintaining information over lengthy time steps. LSTM networks may efficiently describe complicated sequential patterns while addressing problems such as the vanishing gradient problem that arises in deep recurrent designs by integrating memory cells and gating mechanisms. LSTM networks excel at comprehending and exploiting temporal relationships within sequential data, enabling them to discern subtle patterns and dependencies over extended periods. This proficiency is particularly advantageous in SEI applications, where understanding the temporal dynamics of signals is essential for accurate identification and classification. When combined with CNN layers, LSTM networks further enhance the efficacy of pattern recognition and feature extraction in drone signal classification tasks. CNN layers excel at extracting spatial features from signal data, while LSTM layers excel at modeling temporal dependencies. By leveraging the complementary strengths of both architectures, neural network models can effectively capture spatial and temporal characteristics inherent in emitter signals. SEI systems can reliably and precisely identify emitters in a variety of surroundings and signal circumstances thanks to this synergistic integration. As a result, the combination of CNN and LSTM layers provides a strong foundation for improving passive drone identification. The final fused composite feature vector consists of the manual and automatic feature vectors and can be represented by (11).

(11) $${\vec{x}}_{f}\left(n\right)=\left[{\vec{x}}_{\mathrm{mf}}\left(n\right),{\vec{x}}_{\mathrm{af}}\left(n\right)\right]$$

where ${\vec{x}}_{\mathrm{af}}\left(n\right)$ is the automatic feature extracted by the convolutional and LSTM layer of the other branch.

### 2.3. Architecture

**Figure 3:**
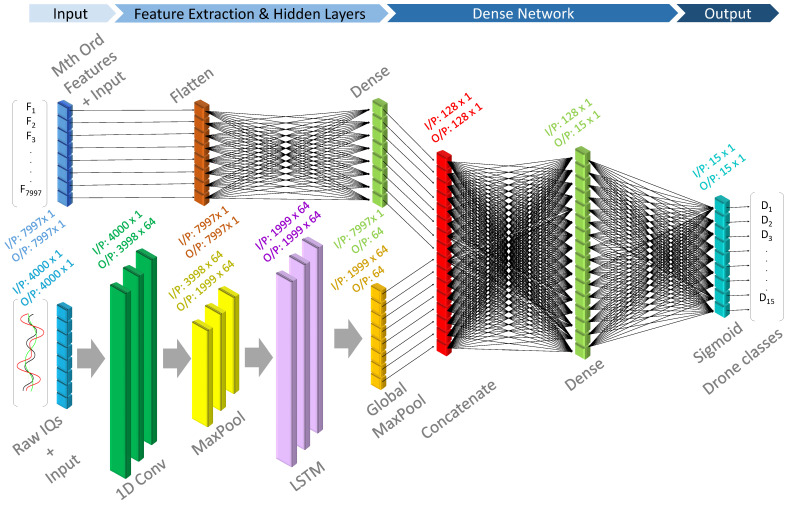
Composite ensemble learning network architecture.

Our suggested network architecture, depicted in Figure 3, is intended to handle two different kinds of input data: manually retrieved features from the raw signal and raw signal data for automatic feature extraction.

A one-dimensional convolutional layer with 64 filters and a kernel size of 5 is applied to the raw signal input. After this layer has extracted features from the raw signal data, the output is down-sampled using a max-pooling layer to reduce spatial dimensions while preserving the most important information. Conversely, the mth order differential characteristics are sent directly to the flattening layer. Following flattening, the input is fed into a dense layer with 64 units and an activation function of the Rectified Linear Unit (ReLU), which extracts features from a manually engineered feature vector. Following that, the outputs from the dense layer and the convolutional layer are combined. The temporal sequence of the data are preserved by passing this concatenated output via an LSTM layer with 64 units, with return sequences set to True. To extract the most pertinent characteristics and reduce the dimensionality of the data, a global max-pooling layer is applied. To obtain the final output predictions, the concatenated and pooled output is then run through a second dense layer that uses a softmax activation function. The sparse categorical cross-entropy loss function, displayed in (12), is used to create the model.

(12) $$L_{\mathrm{CE}}=-\frac{1}{N}\sum_{i=1}^{N}\sum_{j=1}^{C}y_{ij}\log\left(p_{ij}\right)$$

where $p_{ij}$ is the expected probability that sample *i* belongs to class *j*, *N* is the number of samples in the batch, *C* is the number of classes, and $y_{ij}$ is a binary indicator of whether class *j* is the proper classification for sample *i*. Using the supplied labels, training accuracy metrics, and the Adam optimizer, the model is trained for 50 epochs and 64 batch sizes on the unprocessed signal data.

### 2.4. Computational Complexity

To calculate the Floating-Point Operations (FLOPs) for the network, we need to consider the number of arithmetic operations performed during the forward pass. Here is how we calculate it:

**Conv1D Layer (conv1):**
  - Complexity: O(4000×5×64).
**MaxPooling1D layer (pool1):**
  - Complexity: negligible compared to other layers.
**Flatten layer (flatten2):**
  - Complexity: negligible compared to other layers.
**Dense layer (dense1):**
  - Complexity: O(7997×64).
**LSTM layer (lstm1):**
  - Complexity: $O(2000\times 64^{2})$.
**GlobalMaxPooling1D layer (lstm1_pooled):**
  - Complexity: negligible compared to other layers.
**Concatenate layer (merged):**
  - Complexity: negligible compared to other layers.
**Dense layer (dense1):**
  - Complexity: O(128×15).
**Total complexity:**
  - The total computational complexity we obtain is approximately O(9984448).

## 3. Dataset

The dataset [38] that we used consists of RF signals that are intercepted and recorded by a passive RF surveillance system from Radio Controllers (RCs) of different drones. Each drone RC in the collection has about 1000 RF signals, each lasting 0.25 ms and holding 5 million samples. The receiver used a 20 MHz sampling frequency. The collection includes drones from eight different manufacturers, for a total of fifteen different RC models. Interestingly, a variety of signals are produced by drone models that are represented by various RCs. The 2.4 GHz band is used by all RCs to transfer signals, allowing for a thorough investigation of the RF communication protocols these drones employ. Scripts are provided that define a class for drone remote control object creation and functions for data visualization and example feature extraction from the raw radio frequency signal to aid in analysis and understanding. A database of RC objects and metadata are also included in the collection; this is shown in Table 1, and includes details like manufacturer, model, raw RF signal, sampling frequency, and more.

## 4. Experiments, Results, and Analysis

### 4.1. Loss Function and Curves

**Figure 4:**
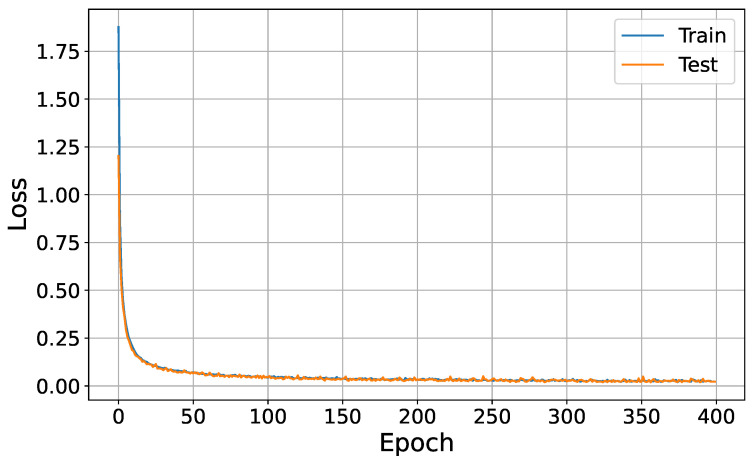
Model training curve.

The curve depicted in Figure 4 provides insightful observations regarding the training and generalization behavior of the model. Initially, the training score curve exhibits fluctuations, indicating active learning from the training data. However, beyond epoch 120, the curve stabilizes, suggesting that the model has effectively captured the underlying patterns within the training data.

Meanwhile, the test score curve closely tracks the training score during the early epochs, demonstrating the model’s ability to generalize to unseen data. As training progresses, the test score curve converges and aligns closely with the training curve, indicating stable generalization performance. This convergence post-epoch 50 signifies that the model has struck a balance between minimizing the loss of the training data and effectively generalizing to new, unseen instances.

### 4.2. SNR vs. Accuracy

This section covers the performance-based analysis of the proposed method on open-source drone remote control radio frequency signal dataset [38].

A detailed and comprehensive analysis of each of the following methods has been carried out:

- Time Frequency Convolutional Neural Networks—Short Time-Frequency Transform (TF-CNN (STFT)) [39].
- Time Frequency Convolutional Neural Networks—Discrete Wavelet Transform (TF-CNN (DWT)) [39].
- Time domain Inphase and Quadrature Convolutional Neural Networks—(T-CNN) [40].
- Dense Network (DenseNet-18) [41].
- Visual Geometry Group (VGG16) [42]

**Figure 5:**
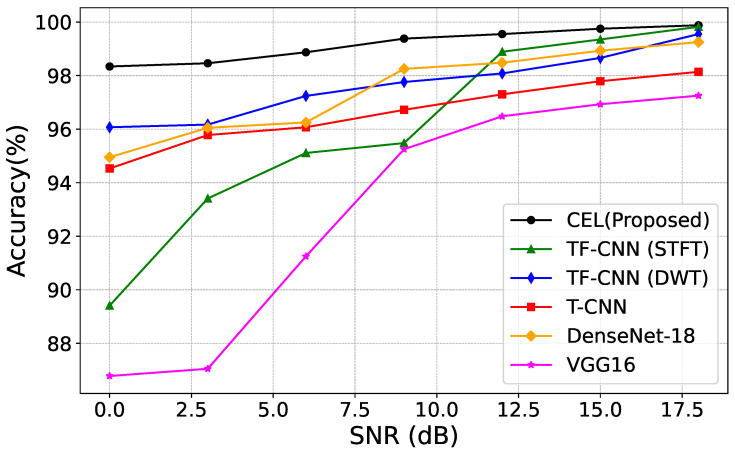
Comparison with existing methods on drones dataset.

Figure 5 compares the classification accuracy and the model’s performance with different SNR levels of the dataset and, thereby, proves the efficiency of the new CEL approach in the drone dataset compared to benchmark deep learning algorithms. The objective of this study is therefore to assess the performance of the proposed CEL method against others under different SNR levels. The CEL method yields a higher recognition percentage than the foregoing methods, especially at a lower SNR of 5dB and below. In situations where other techniques do not allow extracting relevant features from RF fingerprints due to low SNR, the CEL method demonstrated better results that are more stable and less sensitive to increased noise levels.

The significant improvements in the results are mainly attributed to the introduction of denoising procedures within the deep learning system. The wavelet-based denoising is chosen because it performs well with noise present in different frequency bands when the signal is decomposed to different scales. This method unaltered the major signal features while restraining only the noise, which makes it more effective in environments with low SNR and helps to improve the model’s performance. Therefore, we can state that the CEL model can achieve high accuracy as compared to the benchmark techniques, even in high noise conditions.

### 4.3. Batch Size vs. Performance Metrics

**Figure 6:**
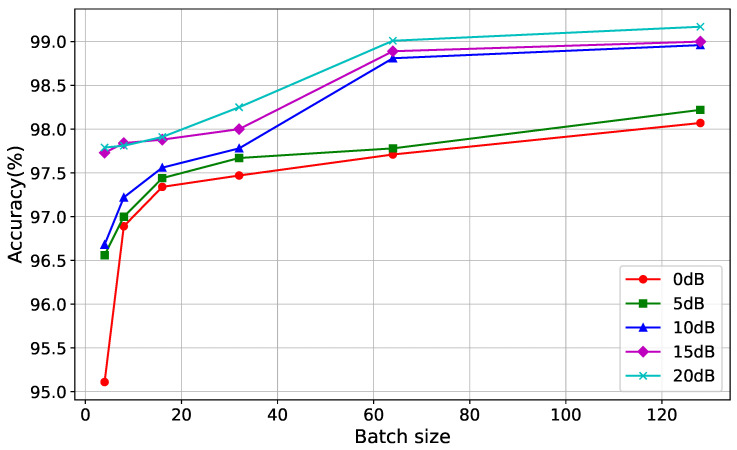
Impact of batch size on classification accuracy.

In Figure 6, we illustrate the influence of batch size on classification accuracy across different SNR values. Our analysis revealed that when the batch size exceeds 15, the achieved accuracy consistently remains above 97.5%. Furthermore, as the SNR surpasses 10 dB, we observed a sharp increase in accuracy, reaching approximately 99%.

These findings underscore the importance of batch size selection in training neural networks for classification tasks, particularly in scenarios with varying levels of noise. Larger batch sizes tend to yield higher accuracies, with notable improvements observed at higher SNR levels. This suggests that larger batches facilitate more stable updates, leading to enhanced model performance.

Table 2 provides the model’s performance for different values of batch size. The evaluation is based on four key metrics, including F1-score, precision, recall, and accuracy. In general, an increase in the batch size leads to the improvement of the model resulting in the growth of all the values. This implies that a larger batch size helps in improving the model’s generalization and, therefore, accuracy. However, the additional analysis, involving statistical tests and graphical analysis, would be required to state more definite conclusions on the influence of the batch size used for model building.

### 4.4. Confusion Matrix

**Figure 7:**
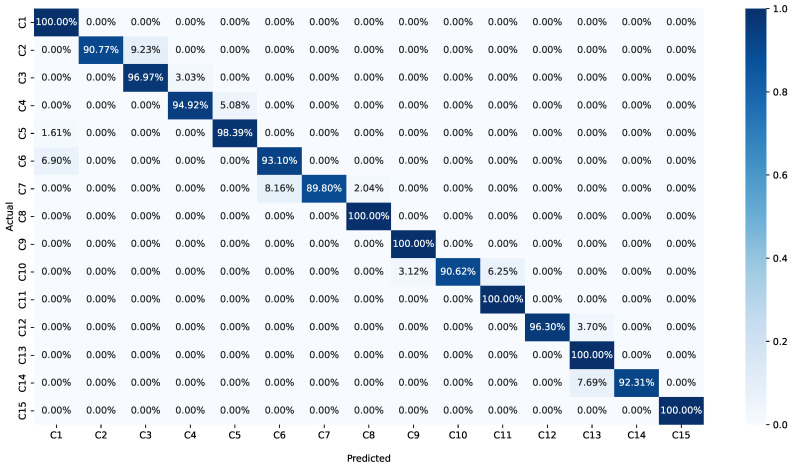
Confusion matrix.

Figure 7 displays the confusion matrix generated by the proposed method, providing valuable insights into its classification performance. The diagonal entries of the matrix represent the number of correct classifications for each class, showcasing the model’s accuracy on individual categories. Meanwhile, the upper and lower triangulation matrices indicate instances of misclassification, offering a clear view of the errors made by the method.

Through rigorous evaluation with 100 iterations of the drone identification method, we observed promising results. When the SNR is greater than 5 dB, the average accuracy of the proposed method surpasses an impressive 98%. This signifies the method’s effectiveness in correctly identifying drone signals under favorable SNR conditions that are above 7 dB SNR. The high accuracy achieved in these experiments highlights the potential of the proposed approach for real-world applications, where accurate identification of drone signals is crucial for communication security and management.

### 4.5. 3D Scatter Plot

**Figure 8:**
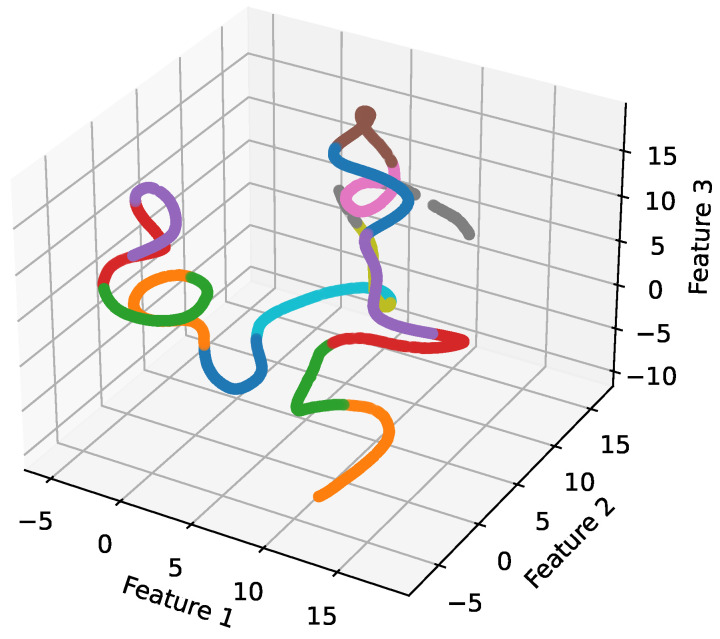
Scatter plot of first three PCA features.

In Figure 8, we present a three-dimensional scatter plot featuring 15 distinct classes and three Principal Component Analysis (PCA) features. Each point in this plot represents an instance from one of the 15 classes, positioned in a three-dimensional space defined by the PCA features. These features serve as axes, with each one representing a principal component, a linear combination of the original features selected to capture maximum data variance.

Notably, the plot reveals clear and distinct regions for each class, showcasing the effective separation of classes within the three-dimensional feature space. This indicates that the PCA features successfully capture the inherent structure of the data, facilitating precise differentiation between classes as driven by the proposed methodology. The reference to “excellent bifurcation capabilities” highlights the discriminative power of both manually crafted and automatically derived features within our methodology. This distinction enables the method to effectively bifurcate classes, demonstrating its ability to discern essential characteristics and patterns that set each class apart. As a result, the scatter plot displays well-defined separation boundaries, underscoring the efficacy of our novel approach.

### 4.6. Box Plot

**Figure 9:**
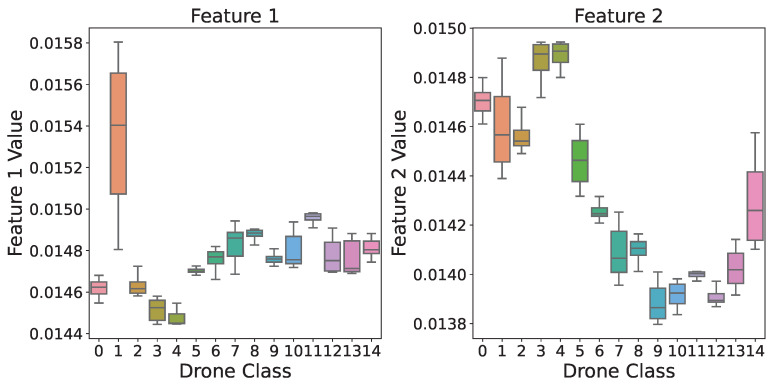

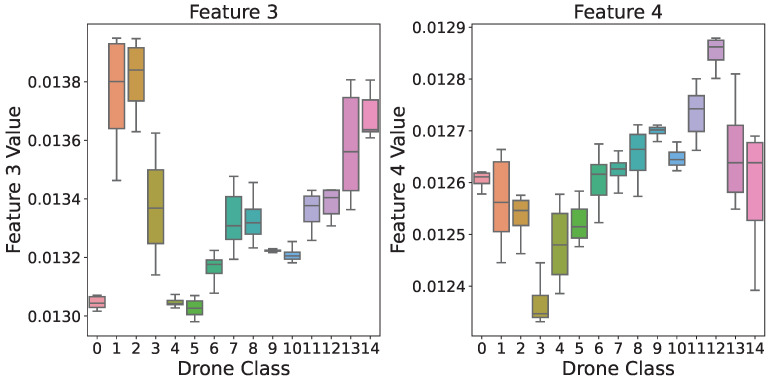
Box plot of first four PCA features.

We examine a box plot in Figure 9 that illustrates the distribution of data points among four Principal Component Analysis (PCA) characteristics in 15 different classes. The plot’s boxes, each of which represents a distinct class, show how the data are distributed among the four PCA features within each class. The middle line in each box represents the median value; the box itself extends from the first to the third quartile (Q3) of the data distribution, providing information on the interquartile range. Moreover, the whiskers extend to outline the data range, eliminating any outliers, which are shown as single dots.

This box plot not only facilitates an understanding of the variability and spread of data within each class, but also offers a glimpse into the effectiveness of our fingerprint features. By analyzing the distribution and spread of data across the PCA features, we gain valuable insights into the distinctiveness of each class and the discriminatory power of our selected features.

Moreover, this plot serves as a complement to the earlier visualization in Figure 8, which showcased the distinct regions occupied by different classes in a three-dimensional space. Together, these visualizations provide a comprehensive understanding of the relationships between the PCA features, class distributions, and the overall effectiveness of our proposed methodology in capturing and distinguishing between different classes.

## 5. Discussion

In this study, we introduce a novel approach for the classification of drones based on RF signals, with the objective of enhancing the accuracy of drone identification under practical conditions. This aligns with the evolving trends in drone technology and its integration into future 6G networks. Our methodology effectively combines handcrafted features with automatic feature extraction through deep learning, amalgamating these features into a single vector that encapsulates a comprehensive array of discriminant information. Consequently, our work synergizes conventional signal processing techniques with advanced deep learning architectures to address the challenges of drone classification, thereby bolstering wireless security in 6G networks.

A pivotal aspect of our approach is its applicability under spread-spectrum techniques, including frequency-hopping spread spectrum. These techniques can distort the original RF fingerprint, potentially misrepresenting the true signal characteristics if the capture period is shorter than the duration of frequency hopping or code spreading. To mitigate this issue, it is imperative that the capture period is sufficiently long to encompass at least two cycles of the modulation. This ensures the preservation of relevant signal variations essential for accurately capturing and characterizing the RF fingerprint. Future research will delve deeper into this aspect.

Our experimental results and the evaluation of various metrics underscore the superiority of the proposed methodology. The trained model demonstrates high accuracy, particularly under conditions of high SNRs. Additionally, our approach exhibits robustness to noise across all SNR levels, including very low SNRs, due to the incorporation of signal denoising techniques within the framework. These findings affirm the efficacy of our method in addressing drone identification challenges across diverse environments and signal conditions, consistent with the requirements of 6G technology.

Moreover, the employed methodology utilizes three-dimensional scatter and box plots, which provide insightful visualizations for estimating the specificity of classes within the feature space. Such representations validate the capability of our approach to accurately classify drone signals based on their RF characteristics and to distinctly delineate different classes.

In summary, this study presents an innovative and forward-looking model for the categorization of drones based on RF signals, contributing significant advancements in the passive identification of drones. This research underscores the critical importance of advanced drone identification techniques in ensuring secure and regulated drone operations within the context of the 6G era.

## 6. Conclusions

In this paper, we unveil Composite Ensemble Learning (CEL), an innovative ensemble learning methodology tailored for drone classification via RF signals.The CEL methodology exemplifies an innovative approach that effectively integrates manual feature engineering with deep learning-based automatic feature extraction, representing a significant advancement from traditional techniques in the field of drone RF signal analysis. Through rigorous experimentation, we have substantiated CEL’s efficacy and resilience in low SNR conditions. We were able to achieve classification accuracy above 98%, even at 0 dB. The visualization techniques employed furnish compelling evidence of CEL’s prowess in discerning intricate patterns within RF data. Our contribution not only pushes the boundaries of passive drone identification techniques, but also holds significant promise for addressing emerging challenges in the realm of 6G technology. By providing a robust solution for drone classification leveraging RF signals, our research offers practical avenues for fortifying security and safety measures amidst the evolving landscape of 6G networks.

## Figures and Tables

**Table 1:** Metadata for the dataset [38].

Maker	Model	Count	Duration
DJI	Inspire 1 Pro	1000	0.25 ms
DJI	Matrice 100	1000	0.25 ms
DJI	Matrice 600	1000	0.25 ms
DJI	Phantom 4 Pro	1000	0.25 ms
DJI	Phantom 3	1000	0.25 ms
Spektrum	DX5e	1000	0.25 ms
Spektrum	DX6e	1000	0.25 ms
Spektrum	DX6i	1000	0.25 ms
Spektrum	JR X9303	1000	0.25 ms
Futaba	T8FG	1000	0.25 ms
Graupner	MC32	1000	0.25 ms
HobbyKing	HK-T6A	1000	0.25 ms
FlySky	FS-T6	1000	0.25 ms
Turnigy	9X	1000	0.25 ms
Jeti Duplex	DC-16	1000	0.25 ms

**Table 2:** Batch size vs. model performance metrics.

Batch Size	F1	Precision	Recall	Accuracy
8	0.97	0.97	0.97	96.56
16	0.98	0.98	0.98	97.89
32	0.98	0.98	0.98	98.22
64	0.97	0.97	0.97	97
128	0.98	0.98	0.98	98.33
256	0.99	0.99	0.99	99

## Data Availability

Data is contained within the article.
